# The Life Cycle and Immature Stages of *Kallima albofasciata*, the Endemic Oakleaf, in the Andaman Islands (Indian Ocean, Bay of Bengal)

**DOI:** 10.1673/031.012.6601

**Published:** 2012-05-22

**Authors:** Veenakumari Kamalanathan, Prashanth Mohanraj

**Affiliations:** National Bureau of Agriculturally Important Insects, P.O. Box 2491, H.A. Farm Post, Hebbal, Bangalore, 560 024, India

**Keywords:** host plant, *Strobilanthes glandulosus*

## Abstract

*Kallima albofasciata* Moore 1877 (Lepidoptera: Nymphalidae), a species of Oakleaf butterfly reported for the first time from the Andaman Islands in 1874, was recognized as an insular endemic in 1877. Studies so far indicate that it is restricted to the contiguous islands of South and Middle Andamans. On these islands it apparently has a very localized distribution, giving rise to fears that it may be vulnerable and could face the threat of extinction with increasing developmental pressures on its habitat. Though it was reported to be extinct in 1993, its presence has since been documented and is currently protected by law in India. Nevertheless, nothing is known about the immature stages, its larval food plants or life history, and very little is known about its habitat and periods of occurrence on the islands. Here, details of all these aspects of this little—known butterfly of the Andaman Islands are presented. This should prove useful in the formulation of a conservation strategy for this iconic Oriental butterfly.

## Introduction

*Kallima* Doubleday 1849 (Lepidoptera: Nymphalidae) is an Oriental genus of Oakleaf butterflies. They are distributed from the Sundaland region in the south to Central China in the north (Corbet and Pendlebury 1992). The genus has generated considerable interest among both entomologists and the lay public, as the adult butterflies resemble dried leaves so closely that it becomes difficult to locate them in their habitats when they are at rest with their wings closed. In a memorable passage in *The Malay Archipelago*, Wallace (1962) details his travails in pursuing *Kallima paralekta* (Horsfield), which played tricks with his eyes as the many attempts that he made at capturing them proved futile and they disappeared from his sight each time they rose into the air when disturbed and settled with their wings folded amongst the foliage.

The endemic Andaman Oakleaf, *Kallima albofasciata* Moore 1877 (Lepidoptera: Nymphalidae), is the only species in the genus *Kallima* known to occur in the Andaman Islands. It is not found in the Nicobars, even though all the land masses around the Nicobars harbor species in the genus.

*Kallima albofasciata* was recognized as a distinct species and described by Moore (1877), though Hewitson (1874) had earlier reported *Kallima* as occurring on the Andaman Islands under the name *K*. *philarchus* (Westwood). *Kallima albofasciata* has been listed as ‘rare’, having only been collected so far from the contiguous islands of Middle and South Andaman (Ferrar 1948). It is protected under Schedule II, Part II of the Indian Wildlife (Protection) Act of 1972. Ferrar (1948) collected them in June—July with a single female being captured in October.

The life cycle of the first Indian *Kallima* to be studied was *K. horsfieldi* from southern India (Davidson et al. 1890), in which the late larval instar and pupa were briefly described. Shortly thereafter Davidson et al. (1896) provided illustrations of these two stages. Meanwhile, a more detailed description of all the immature stages (including the egg) as well as the durations of each of the stages for the North Indian *K. inachis* was furnished by Dudgeon (1895). The most detailed descriptions of the egg, the final larval instar, and the pupa for *K. horsfieldi* resulted from the meticulous studies of the life cycles and immature stages of Indian butterflies by Bell (1910). Very recently (Balakrishnan 2010) photographed all the pre—imagines and detailed the life cycle of *K. horsfieldi.* The study does not, however, give the durations of each of the larval instars, but instead gives the combined duration of all the instars.

The immature stages and the life cycle of *K. albofasciata* have not been detailed so far— with the exception of photographs of the egg, late larval instar, and pupa by Veenakumari et al. (2008). This study details the life history and describes the immature stages of this Oakleaf butterfly.

### Habitat characteristics

Eggs and larvae of *K. albofasciata* were collected from the moist deciduous forest of Chiriyatapu (11° 13′ N, 92° 42′ S) at the southern extremity of South Andaman Island. These forests are on low hilly ground not exceeding 100 m in altitude. Immature *Kallima* were found on *Strobilanthes* shrubs growing close to the ground between the bases of tree trunks. This region of the forest was close to the shoreline, which was fringed with intermittent stands of mangroves.

South Andaman experiences a tropical maritime climate and receives both the southwest and northeast monsoons between May and December. The mean minimum and maximum temperatures vary from 23 °C to 30 ° C, with maximum temperatures reaching ∼34 °C in April. High humidity prevails throughout the year, ranging from ∼60–90%.

## Materials and Methods

Field surveys were regularly undertaken in both the Andamans and the Nicobars during 1989–2000. Censuses were carried out not only to record adult butterflies, but also to look for the eggs and other pre—imagines of butterflies inhabiting these islands to determine their larval host plants, learn to recognize their immature stages, determine their periods of occurrence, and study their life cycles. All immatures encountered in the field were brought to the laboratory along with their food plants to rear a number of them.

The field collected immature stages (eggs/larvae) were confined individually in plastic containers of suitable sizes. They were attended to daily between between 9 AM – 12 PM. The rearing containers were cleared of larval excrement as well as post—feeding remnants of leaves and shoots. They were then wiped clean with a dry cloth and the larvae placed back in their respective containers with a fresh supply of host plant leaves.

All observations were taken when the rearing containers were cleaned. The containers were also carefully examined at this time for the presence of head capsules to determine the duration of each instar. Measurements were taken with a pair of dividers and descriptions were made under a stereobinocular microscope. Neither head capsules nor larvae were preserved.

## Results

### Habitat and larval host plant

An egg and larvae of *K. albofasciata* were found on leaves of *Strobilanthes glandulosus* Kurz (Acanthaceae) at Chiriyatapu, along the southeastern coast of the island of South Andaman. The plants formed part of the understory of the moist deciduous forest that is characteristic of this part of this island. The plants grew along the lower slope of a hillside forming one face of a valley. A shallow, seasonal stream flows along the floor of the valley, which was dry during April±—May when the preimagines of *K. albofasciata* were found.

The *S. glandulosus* plants were distributed alone or in pairs about 3–4 feet apart. The majority of them had put forth a new flush of leaves. Many had flowered and set fruit. While a few plants retained the pale blue tubular flowers, the others had dry, dehisced capsules.

### Pre—imaginal stages in field

One egg had been laid on the dorsal surface at the tip of a tender leaf. The early instars were found on the ventral surfaces of tender leaves. They ate the leaves from the margins towards the midrib. The midrib and the bases of the major veins were left uneaten with larvae resting on the undersurface, facing away from the petioles.

Older instars fed on the older leaves, which were thicker and rougher. They also fed on most of the midrib except for its base. They left the petioles uneaten. These larvae were found along the main stem of plants, close to the base and faced away from the ground.

### Life cycle

The total life cycle was completed in 37–47 days. The incubation period was about five days. The larvae passed through six instars in 23–32 days with the sixth instar taking 8–11 days. The pupal period varied from 9–11 days (Table 1).

### Description of pre—imaginal stages

**Egg.** Laid singly. Green, spherical, with white longitudinal ribs. Turns whitish when approaching the time for hatching (Figure 3).

**Larvae. First instar**: Measurement: length = 0.2 cm, n = 2. Pale green or cream with a glossy black head. Head bare, devoid of horns. Very small cream hairs are present. Yellow warts on prothoracic and anal segments. Black dorsal warts on the metathorax and on the first and second abdominal segments. Setae on warts black. **Second instar**: Measurement: length = mean 0.55 cm, range 0.5–0.6 cm, n = 2. Overall black in color with a pair of spines on head that are terminally clubbed with short sparse spines along its length and are present in all the succeeding instars. The spines are black and branched along the length of the body. **Third instar**: Measurement: mean length 1.0 cm, range 0.8–1.2 cm, n = 3. Black in color like the previous instar, but two rows of sub— dorsal silver spots are present for the first time. **Fourth Instar**: Measurement: mean length 1.64 cm, range 1.4–1.8 cm, n = 7. Overall black, similar to previous instar with two rows of silver spots numbering ten in each longitudinal row. **Fifth Instar**: Measurement: mean length 2.2 cm, range 1.7– 2.7 cm, SD = 0.32, n = 10. Velvety black larva with two large branched horns on head. **Sixth Instar**: Measurement: mean length 3.74 cm, range 2.8–4.5 cm, SD = 0.51, n = 16. Remains velvet black, but the spines were reddish in color (Figure 4). As the larva aged the reddish color became more intense. Dorsally the larva had a longitudinal ashy—white band and a brown lateral band with yellow spots along the sides.

**Table 1.**
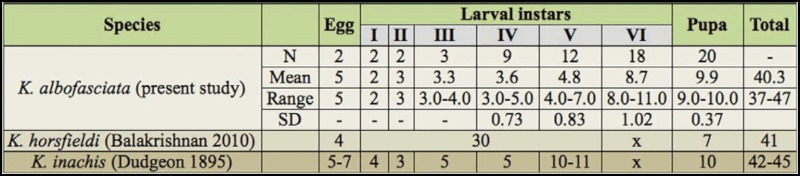
Life cycle of *Kallima albofasciata* compared with the life histories of Indian species of *Kallima* for which the life cycles are known (durations in days).

**Figures 1–5.**
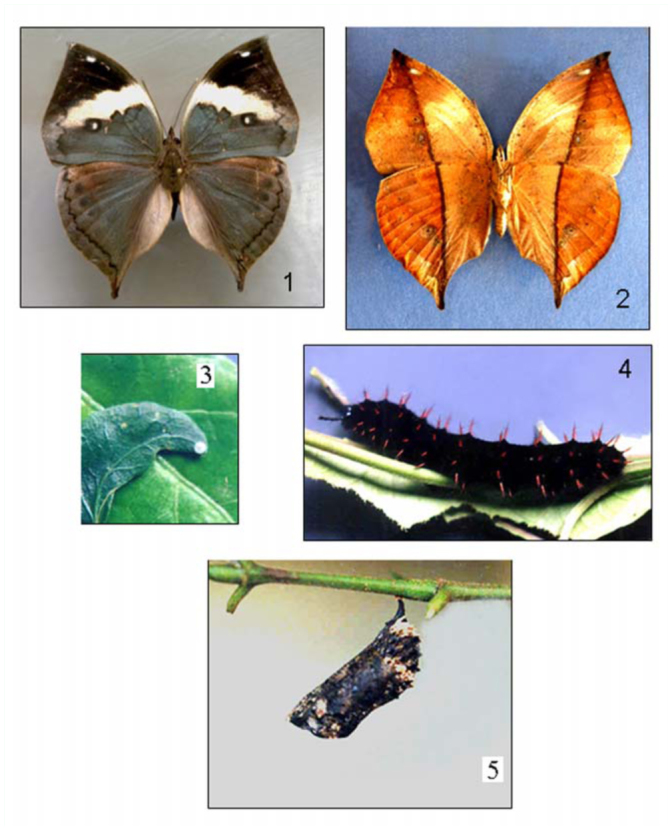
*Kallima albofasciata*: (1) adult — dorsal surface; (2) ventral surface; (3) egg; (4) sixth larval instar; (5) pupa. High quality figures are available online.

**Pupa.** Measurement: mean length 2.7 cm, range 2.0–3.1 cm, n = 8; width = mean 0.79 cm, range 0.70–0.90 cm, n = 8. Pupates head downwards, anchored by a sturdy cremaster. The pupa was brown with dirty white dorsal bands on abdomen and spots of similar color towards the anterior end (Figure 5). When freshly formed pupae were greenish towards the anterior extremity which gradually turned a dull brown—black as it aged.

## Discussion

*Kallima albofasciata*, *K. horsfieldi*, and *K. alompra* are all protected under Schedule II Part II of the Indian Wildlife (Protection) Act of 1972 (Anon 2005). *Kallima inachis* is the only Indian species that is not protected by the Act. In spite of its protected status, no attempts have been made to study the life cycles, larval food plants, immature stages, or the island—wise distribution of *K. albofasciata.*

A breeding population of *K. albofasciata* was found at Chiriyatapu on the island of South Andaman between the last week of April and the first week of May 2000. Soubhadra Devy et al. (1998) also recorded the species in their study conducted during the years 1992 and 1994, although it had been listed as extinct in the Andamans (Khatri 1993).

*Strobilanthes glandulosus* was found to be the larval host plant of *K. albofasciata*. This plant is endemic to the Andaman Islands (Vasudeva Rao 1986), but other species of *Strobilanthes* are known to be the larval food plants of other species of *Kallima* (Davidson et al. 1890; Dudgeon 1895; Corbet and Pendelury 1992; Balakrishnan 2010). Species of the genus *Pseuderanthemum* are also known to be larval host plants of *Kallima* (Corbet and Pendlebury 1992). *Pseuderanthemum album* and *S. glandulosus* are present in both the Andamans and Nicobars (Vasudeva Rao 1986; Veenakumari et al. 1997). In addition to the presence of these favored food plants of *Kallima* in the Nicobars, this butterfly occurs on all the land masses surrounding the Nicobars, though it has not yet been recorded on the islands. An intensive search could reveal the presence of this butterfly (perhaps a different subspecies) in the southern Nicobars.

Based on eight years of study on the butterflies of these islands Ferrar (1948) found that *Kallima* were found in June, July, and occasionally in October. However, our study showed a well represented population in April and early May. The monsoon does not commence on the islands during this period. This is a dry period, while June, July, and October are months that receive considerable rainfall. Nevertheless, observations from our study showed that the *S. glandulosus* stand, which supported the larvae of *K. albofasciata*, had put forth a new flush, and the tender leaves appeared to be the preferred egg laying site as well as the food of early instars.

Both *K. horsfieldi* and *K. inachis* have been found to pass through five larval instars in India (Dudgeon 1895; Balakrishnan 2010). *Kallima albofasciata*, on the other hand, has six larval instars (Table 1). In China, *K. inachis* was found to generally pass through five larval instars, although there were less common instances of individuals having six larval instars.(Yang Ping et al. 2005).

There are differences in larval coloration between *K. albofasciata* and the other Indian species. For instance, the first instar of *K. albofasciata* is cream—colored, while the other Indian species are brownish in color (Dudgeon 1895; Bell 1910; Balakrishnan 2010). The silver spots that we noticed in the third and fourth instars in *K. albofasciata* are not described for the other Indian species. The morphologies of all these species have to be studied in greater detail to establish other differences. Molecular studies will also help establish the specific status of each of these species.

If *K. albofasicata* is localized and restricted in its distribution as it is now perceived, then the ‘developmental’ activities and the expanding human population on the islands could make it highly vulnerable to extinction. It is interesting to note in this context that while Ferrar (1948) listed it as ‘rare’, Moore (1899– 1900) stated that Roepstorff (a Danish administrator in the service of the British and a collector of insects) took ‘numerous examples of this species’ from Port Blair on South Andaman. Roepstorff collected in the 19^th^ century shortly after the British had annexed the islands, while Ferrar collected almost a century after the islands were colonized. It is tempting to conclude that a century of colonization (which entailed considerable clearing of forests and logging on the islands of South and Middle Andaman) adversely impacted localized populations of butterflies such as *K. albofasciata.*

Studies ought to be initiated to determine the present distribution and status of *K. albofasciata* in the Andaman Islands. Surveys should also be undertaken in the South and Central Nicobars to establish the presence or absence of these unique butterflies that, as Wallace (1962) stated, sport “the most singular and most perfect disguise…ever met with in a lepidopterous insect…”.
